# Current status of laboratory and imaging diagnosis of neonatal necrotizing enterocolitis

**DOI:** 10.1186/s13052-018-0528-3

**Published:** 2018-07-25

**Authors:** Gabriella D’Angelo, Pietro Impellizzeri, Lucia Marseglia, Angela Simona Montalto, Tiziana Russo, Ignazio Salamone, Raffaele Falsaperla, Giovanni Corsello, Carmelo Romeo, Eloisa Gitto

**Affiliations:** 1 Department of Clinical and Experimental Medicine, University of Messina, Via Consolare Valeria, 1, 98125 Messina, Italy; 2 Neonatal and Pediatric Intensive Care Unit, Department of Human Pathology in Adult and Developmental Age “Gaetano Barresi”, University of Messina, Via Consolare Valeria, 1, 98125 Messina, Italy; 3 Unit of Pediatric Surgery, Department of Human Pathology in Adult and Developmental Age “Gaetano Barresi”, University of Messina, Messina, Italy; 4 Oncological Radiology Unit, Department of Biomedical and Dental Sciences and Morphofunctional Imaging Policlinico G. Martino Hospital, University of Messina, Messina, Italy; 5 General Pediatrics and Pediatric Acute and Emergency Unit, Policlinico-Vittorio-Emanuele University Hospital, University of Catania, Catania, Italy; 6 Department of Maternal and Child Health, University of Palermo, Palermo, Italy

**Keywords:** Necrotizing enterocolitis, Diagnosis, Biomarkers, Imaging, Newborn

## Abstract

Necrotizing enterocolitis continues to be a devastating disease process for very low birth weight infants in Neonatal Intensive Care Units. The aetiology and pathogenesis of necrotizing enterocolitis are not definitively understood. It is known that necrotizing enterocolitis is secondary to a complex interaction of multiple factors that results in mucosal damage, which leads to intestinal ischemia and necrosis. Advances in neonatal care, including resuscitation and ventilation support technology, have seen increased survival rates among premature neonates and a concomitant detection in the incidence of this intestinal disease.

Diagnosis can be difficult, and identifying infants at the onset of disease remains a challenge. Early diagnosis, which relies on imaging findings, and initiation of prompt therapy are essential to limit morbidity and mortality. Moreover, early management is critical and life-saving.

This review summarizes what is known on the laboratory and instrumental diagnostic strategies needed to improve neonatal outcomes and, possibily, to prevent the onset of an overt necrotizing enterocolitis.

## Background

Necrotizing enterocolitis (NEC), a condition characterized by intestinal necrosis affecting the ileum, jejunum and/or colon, is a leading cause of morbidity and mortality among premature infants [1]. The term “necrotizing enterocolitis” first appeared in the 1950s when Schmid and Quaiser [2] described infants dying from necrotic lesions of the gastrointestinal tract. However, it was not until the 1960s, when Barlow et al. [3] reported a series of 64 infants with NEC that it became recognized as a distinct clinical entity. The incidence varies from 0.5 to 5 per 1000 live births, but NEC is predominantly a disease of very low birth weight (VLBW) infants, with a clinical picture of focal intestinal perforation, a distinct subset of NEC in terms of surgical management [4, 5]. Preterm newborns have many intestinal vulnerabilities that permit microbial pathogens to invade tissue and cause severe morbidity and mortality [6]. Animal models of NEC have shown that the innate immune system and, specifically, toll-like receptor-4 are crucial components of NEC vulnerability. This receptor recognizes lipopolysaccharide and is thus a potent sensor of pathogenic bacteria overgrowth and translocation across the mucous barrier of the mucosa [7]. Its pathogenesis is not yet completely understood; several risk factors have been reported, such as immaturity, ischemia, altered gut microflora, decreased mucin barrier, increased gut permeability, reduced gut immunity, and type of enteral feeding formula [8–11]. This multifactorial aetiology leads to the one common final pathway of oxidative stress, inflammation, and necrosis of the neonatal bowel [8, 9]. Mortality rates remain high; infants who die of NEC commonly (66%) die quickly, and new therapeutic interventions are needed [7]. Current management includes empiric administration of broad-spectrum antibiotics, initiation of bowel rest, bowel decompression, infusion of fluid and inotropes to support cardiac function [12]. Surgical intervention is required in up to 50% of NEC cases and includes the removal of necrotic intestine, ileostomy or anastomosis [12]. To date, the primary and most promising method of reducing NEC morbidity lies in NEC prevention [13].

This paper summarizes what is known about the diagnostic strategies needed to improve neonatal outcomes and, if possible, to prevent the onset of complicated NEC.

### Laboratory diagnosis

Early and accurate identification of NEC represents the prime objectives of clinical practice, but a truly early diagnosis is limited by the lack of any highly sensitive and prompt tests to detect signs of NEC and to identify newborns who will develop this intestinal condition [13, 14]. Clinical biomarkers have not been reliable thus far in predicting when NEC will occur or who will develop the disease. Several biochemical alterations, such as raised or depressed white cell count, thrombocytopenia, metabolic acidosis, glucose instability and elevated C-reactive protein levels, can be observed in infants affected by NEC [15, 16], but none of these laboratory parameters present accurate sensitivity and specificity. Particularly, it has been studied whether the platelet neutrophil product could be useful in assessing the prognosis of patients with NEC, either in terms of extent of disease or survival. Ragazzi et al. constructed ROC curves to evaluate the relationship between specificity and sensitivity of these products; they showed an area under the ROC curve for survival of 0.58 for neutrophils, 0.75 for platelets and 0.71 for and their product, indicating that these last performed no better than initial platelet count alone as an indicator of survival; however, ROC curves for discriminating between patients with either multifocal or panintestinal involvement, showed that platelet-neutrophil product, with an area under the curve of 0.69, performed better than either platelets alone (0.65) or neutrophils alone (0.64) [15].

Moreover, different classes of components of the inflammatory cascade, such as C-reactive protein, serum amyloid A, chemokines, cytokines, and interleukins, can helpful in accurate diagnosis or predicting the severity and evolution of NEC [17].

Yang et al. investigated the diagnostic value of pre-albumin in neonates with severe NEC, demonstrating that this biomarker could be an important value for the diagnosis of severe NEC (≥IIB) with high sensitivity and specificity [18].

There are also other more specific markers of intestinal damage, such as intestinal fatty acid-binding protein (I-FABP) [19, 20], which is released into the circulation from damaged enterocytes and excreted in urine, but these are not yet suitable for routine clinical use. Liebermann et al. reported that plasmatic levels of I-FABP might serve as a diagnostic marker for early intestinal mucosal compromise, such as NEC [21]. Several studies found plasmatic concentrations of I-FABP to be a specific marker for early identification of severe NEC (Bell’s stage III), but less useful for differentiating initial Bell’s stages [22, 23]. Regarding the potential diagnostic value of I-FABP measured in plasma (I-FABPp) and urine (I-FABPu) for the detection of NEC, Schurink et al. demonstrated that a sensitivities for both I-FABPp and I-FABPu dropped to 11–77% and 5–50% respectively, with specificities soon equalling 100% [24]. Therefore, the specificity for I-FABP to determine whether patients would develop complicated NEC gradually increased over time, ranging from 75% at onset of disease to 91% after 48 h [24]. However, concentration in healthy premature infants is very variable and half-life in plasma is short, so that use of I-FABP concentration in plasma or urine to diagnose early NEC is limited. Therefore, I-FABP can to be used to identify infants that have advanced disease and who might thus require more extensive resection [25]. Besides, the recent multicenter study conducted by Heida et al. demonstrated that both plasma and urine I-FABP levels are strongly associated with the length of bowel resection in newborns with surgical NEC, supporting the hypothesis that increased I-FABP levels correspond with the extent of necrotic tissue [25].

Recently, other innovative biomarkers, such as the urinary proteome [26] and peptidome [27] have been identified and have been related with poor outcome, including disease progression, but these biomarkers alone were not able to distinguish progressors from non-progressors. However, these biomarkers, along with other clinical factors, could predict the clinical subset of NEC [26, 27].

Claudins, tight junction proteins, have been identified as potential biomarkers for inflammatory bowel disease in adult patients as well as for intestinal injury in mouse models. Recently, it has been shown that urinary claudin-2 presented elevated levels in neonates with NEC as compared to those without NEC; therefore, this molecule could represent a potential predictor of early NEC [28].

Based on several investigations, it has been demonstrated that acute intestinal ischemia, reperfusion and bacterial colonization were associated with failure of the mucosal barrier, increasing permeability of the intestinal wall and, thus, allowing D-lactate to enter the portal circulation with increased plasma D-lactate levels in both portal and systemic blood [29]. Previously, Garcia et al. found that urinary D-lactate increased in infants with NEC and demonstrated increased enteric bacterial activity in NEC [30]. Recently, Guofeng et al. have examined levels of plasma D-lactate in premature infants affected by NEC, finding them significantly increased early, in direct proportion to the overall extent of intestinal disease, without specify sensitivity and specificity [31]. Authors hypothesized that the use of plasma D-lactate levels as a marker of intestinal injury could be useful as a diagnosis indicator in the early stage of NEC.

Another test adopted by many NICUs was routine faecal occult blood (FOB), based on the hypothesis that intestinal injury and inflammation associated with NEC would lead to occult blood in the feces [32]. Abramo et al. performed a prospective blinded study testing for occult hematochezia within the first six weeks of life in 95 neonates with birth weights < 1800 g who were receiving at least some enteral feeds. Fifty-eight percent of the infants had one or more positive FOB results during the first six weeks of life, and the incidence of NEC was higher in those infants with negative FOB (9.8%) than in those with positive FOB (3.7%). Therefore, they concluded that occult hematochezia was frequent in this population and did not seem to be related to NEC, demonstrating poor sensitivity, specificity, and predictive values of FOB testing on NEC diagnosis [33]. Lately, Pickering et al. have evaluated the value of routine FOB testing on the identification of VLBW infants with NEC. They demonstrated that positive FOB testing occurred in a majority of VLBW infants, with higher odds in the more preterm and intrauterine growth restriction, but the sensitivity, specificity, and predictive value of routine FOB testing for identifying NEC were all very poor, concluding that this test offers no advantages in the early diagnosis of NEC [34].

To date, to our knowledge, none biomarkers mentioned above present the characteristics of the “ideal biomarker”, that is a well-defined optimal cut-off level, a sensitivity and negative predictive value approaching 1.00, specificity and positive predictive value > 0.85, as well as daily or on-demand availability in routine clinical laboratories [17].

### Imaging diagnosis

Imaging modalities used with neonates during the active phase of NEC include plain abdominal radiography and, recently, abdominal sonography. Radiographic imaging is essential in the diagnosis of NEC. Bell’s clinical staging of NEC was published in 1978 and was a major force that facilitated grouping of NEC into patient cohorts rather than as case reports and series [35]. Bell’s Criteria has been the mainstay for the diagnosis and staging of NEC for the last three decades. However, Bell’s staging has been modified by Walsh and Kliegman [36], who divided each stage into two subcategories, and included signs that differentiate between milder and more severe courses of disease, such as absent bowel sounds, abdominal tenderness and ascites, as well as laboratory parameters indicative of acidosis, thrombocytopenia, neutropenia and disseminated intravascular coagulation [36]. Successively, a new ANID taxonomy, sometimes called Gordon’s classification was ideated [37]; although, Bell Staging continues to be used as the standard of practice to diagnose, stage, and treat NEC [35]. The most important radiographic findings of plain abdominal radiography to confirm diagnosis of NEC are pneumatosis intestinalis, portal venous gas and pneumoperitoneum [38]. Seminal breakthroughs have occurred in this decade, such as ventilation support and total parental nutrition, and with them a “new NEC” has emerged in increasingly premature infants who have survived because of these technologies. Once patients are diagnosed with definitive NEC (Bell’s stage 2), significant intestinal injury is likely to occur. Therefore, it is possible that earlier detection of intestinal injury and appropriate treatment might prevent the progression of this disease [39]. Plain abdominal radiography remains the main diagnostic tool in the diagnosis and follow-up of NEC. However, it is sometimes impossible to expose patients to consecutive episodes of radiation. Ultrasound examination (US) seems to be an alternative to current standard usage of radiography [40]. US is an ideal modality for evaluating bowel necrosis as it is non-invasive, does not involve the use of ionizing radiation and can be performed readily at the bedside. Many studies emphasized the numerous advantages of US over plain abdominal radiography, including no exposure to ionizing radiation, no limitation on frequent use, availability, possibility of use at patient’s bedside, possibility of evaluating indices of bowel dynamics, bowel wall thickness, echogenicity, pneumatosis intestinalis, rate of bowel wall perfusion, ability of determining the nature, and estimating the amount, of intra-abdominal liquid [41, 42], but its role is still underestimated and plain radiology remains the gold standard modality for diagnostic purposes. Pneumatosis intestinalis and portal venous gas are both seen during abdominal US, detected earlier than by plain radiography [43]. Moreover, US permits visualization of the bowel wall in a much more detailed way than is possible with plain radiographs, with an assessment of the thickness and perfusion of the intestinal wall and peristalsis; moreover, other information, such as the presence of free intraabdominal gas and the presence of any free intraabdominal fluid, indicative of intestinal perforation, can be obtained from abdominal US [44]. Ultrasonic findings can also contribute to the prediction of the severity of NEC. Yang et al. investigated the value of abdominal US in diagnosing NEC and its significance in evaluating disease severity [45]. According to the modified Bell-NEC staging criteria, authors enrolled 84 neonates, 44 with suspected NEC and 40 with confirmed NEC; according to clinical prognosis, they were divided into a medical treatment (*n* = 58) and a surgery/death group (*n* = 26), and changes in the results of abdominal ultrasound and abdominal X-ray plain film between groups were compared. In the confirmed NEC group, abdominal US showed significantly higher detection rates of portal venous gas and dilatation of the intestine than abdominal X-ray plain film; compared with the medical treatment group, the surgery/death group had significantly higher detection rates of dilatation of intestine, bowel wall thickening, peritoneal effusion and free intraperitoneal air (*P* < 0.05). Furthermore, dilatation of the bowel and free intraperitoneal air shown by abdominal X-ray plain film were more common in the surgery/death group. Therefore, authors concluded that ultrasonic findings could permit predicting the severity of NEC [45]. In another study, it has been stated that abdominal US and radiography in patients with NEC can help predict the outcome [46].

Until today, it was unclear from scientific literature if bowel US findings could provide useful prognostic information to help guide clinical decision-making. Recently, it has been reported that bowel US may be useful for early identification of high-risk infants who may benefit from surgery treatment [47]. Authors conducted a meta-analysis with the identification of several bowel US characteristics associated with poor outcomes in infants with NEC. It has been demonstrated that bowel US features most strongly associated with surgery or death were free air, absent peristalsis, complex ascites and focal fluid collection; while, other minor features, as bowel wall thinning, increased bowel wall echogenicity, absent perfusion and dilated bowel were correlated with a moderate association with surgery treatment or death. Therefore, all these findings could suggest important addition bowel US that may help identify infants at high-risk for poor outcomes [47].

Pneumoperitoneum, detected by abdominal radiography, is the only sign that has been universally agreed on as an indication for surgery [48]; however, this is not present in all babies with bowel necrosis and perforation. Furthermore, colour Doppler US is more accurate than abdominal radiography in depicting bowel necrosis in NEC [49]. The absence of bowel wall perfusion at colour Doppler US is more sensitive and specific than the presence of free air at abdominal radiography in the detection of necrotic bowel in NEC [49]. Faingold et al. showed that colour Doppler US is more sensitive and specific than abdominal radiography in the detection of necrotic bowel [38]. Abnormal US and radiography findings were the most powerful predictors of the need for surgical intervention, including persistent dilation of the bowel loops and evidence of portal venous gas, which were detected by radiography, and bowel wall thickening, absent peristalsis and echogenic-free fluid or focal fluid collection, which were detected by ultrasonography. Therefore, the presence of both echogenic fluid and focal fluid have been reported to be indicators for surgical intervention [50]. Other studies have also evaluated the use of computed tomography and magnetic resonance imaging, but these modalities have not been found to be useful in clinical practice [51, 52].

## Conclusion

NEC diagnosis most often relies on a combination of clinical symptoms and signs, including feeding intolerance, abdominal distention and bloody stools, and radiological features, such as pneumatosis intestinalis, portal venous gas and pneumoperitoneum [14]. Although improvements in the prevention, diagnosis, and treatment of NEC have been made, the only effective strategy is developing a multidisciplinary programmatic approach to reducing NEC. Personalized treatment strategies would be the ideal, but will not be readily available until “ideal” predictive and diagnostic biomarkers have been discovered. Also imaging diagnosis has evolved. It has been documented that combining abdominal radiography and ultrasonography are, to date, the techniques that together could predict the need for surgical intervention [53].

In conclusion, several studies are needed to discover therapeutic strategies to attenuate uncontrolled inflammatory reaction and provide other medical opportunities to treat NEC, before it evolves into a serious sepsis [54].

## Abbreviations

FOB: Faecal occult blood
I-FABP: Intestinal fatty acid-binding protein
NEC: Necrotizing enterocolitis
US: Ultrasound examination
VLBW: Very low birth weight
